# *In vivo* neutralization of the protagonist role of macrophages during the chronic inflammatory stage of Huntington’s disease

**DOI:** 10.1038/s41598-018-29792-x

**Published:** 2018-07-30

**Authors:** Jeffrey Pido-Lopez, Ralph Andre, Agnesska C. Benjamin, Nadira Ali, Sahar Farag, Sarah J. Tabrizi, Gillian P. Bates

**Affiliations:** Huntington’s Disease Centre, Department of Neurodegenerative Disease and Dementia Research Institute, UCL Institute of Neurology, University College London, London, WC1N 3BG UK

## Abstract

Neurodegenerative diseases, characterised by the progressive and selective neuronal death in the central nervous system, are frequently accompanied by an activated immune system. In Huntington’s disease (HD), clinical and animal studies show evidence of immune activity, along with hyper-reactive monocyte/macrophage responses, while application of immunosuppressive regimens have imparted beneficial effects to HD mice. These findings suggest a contributory role of the immune system in HD pathology, with immune-based interventions offering a potential therapeutic strategy. Herein, we show that peripheral and CNS immune system activity increased with disease progression in HD mouse models and defined the phenotype of the immune response. Additionally, the depletion of monocytes and macrophages *in vivo*, via clodronate liposome treatment, revealed a major contributory role of these innate immune cells to the chronic inflammatory milieu observed during the course of the disease. This suggests that peripheral immunomodulatory strategies targeting monocytes and macrophages could be relevant for HD.

## Introduction

The contribution of the immune system to the pathology of neurodegenerative diseases such as Alzheimer’s disease, Parkinson’s disease and Huntington’s disease (HD) has gathered increasing interest and momentum with the ultimate hope of finding effective immune-based therapeutic strategies^1–5^. HD is an autosomal dominant neurodegenerative disorder characterized by progressive cognitive, psychiatric and motor impairments caused by neuronal dysfunction and cell death. The causative mutation is a CAG trinucleotide repeat expansion in exon 1 of the huntingtin (*HTT*) gene leading to an expanded stretch of 38 or more glutamine residues in the N-terminal region of the HTT protein^6^.

*HTT* is more highly expressed in neurons as compared to other cell types^7^. However, immune cells including B cells, T cells, monocytes and/or macrophages from patients show detectable levels of HTT that are positively correlated to disease burden and brain caudate atrophy^8,9^. While microglial cells, the major component of the central nervous system (CNS) immunity, have long been observed to be activated in presymptomatic HD^10^, more recent results suggest a role for the peripheral immune system in HD progression. This has taken the form of elevated levels of plasma cytokines^9^ and chemokines^11^ such as IL6 and TNFα in HD mouse models and patients, along with dysregulated monocyte and macrophage *in vitro* responses^9,12,13^. Indeed, studies utilising immunomodulatoy regimens in HD mouse models substantiate this premise. Beneficial effects have been obtained through the replacement of the HD immune system with a normal one, via bone marrow transplantation^14^, or by treatment with immunosuppressive agents^15,16^ whose effects have been specifically limited to immune cells outside of the brain. The ability of mutant HTT to promote the activation/signalling of the pro-inflammatory transcription factor NFκB may provide one mechanism for the enhanced monocyte/macrophage cytokine secretions seen in HD^13^.

Knock-in HD mice precisely model the genetic basis of HD. They have been generated by either inserting a highly expanded CAG repeat into the mouse *Htt* gene, e.g. *Hdh*Q150^17^, or by replacing mouse exon 1 *Htt* with a mutated version of human exon1 *HTT* e.g. zQ175^18^. Incomplete splicing of the *Htt* gene in all knock-in models^19^ and HD patient brains^20^ results in the production of a small polyadenylated transcript that encodes the highly pathogenic exon 1 HTT protein. The R6/2 HD mouse line is transgenic for the 5′ region of the *HTT* gene^21^, expresses an exon 1 HTT protein and is a model of this incomplete splicing event. R6/2 mice with approximately (CAG)_200_ and knock-in mice with similar CAG repeat expansions (e.g. *Hdh*Q150 and zQ175) develop highly comparable behavioural, molecular and neuropathological phenotypes^22^, but over different time scales: late-stage disease is at ~14 weeks of age for R6/2 mice and close to two years for the *Hdh*Q150 and zQ175 knock-in lines.

Herein, we assessed HD mouse monocyte, macrophage and other immune cells from blood, brain and/or spleen during early symptomatic and late stage HD for signs of immune activity, as indicated by increased immune cell activation markers and cytokine production. We find dysregulated monocyte/macrophage activity in agreement with previous studies^9,13^, but also detect increased dendritic cell (DC) activation. The major role of macrophages and DCs as antigen presenting cells during T cell activation, coupled with our observation of increases in cytokines that are predominantly secreted by T cells, prompted us to additionally investigate T cell activation during HD. Our results revealed that these cells were also dysregulated and showed an increase in activity during the latter stages of disease. Subsequently, we used clodronate liposomes^23,24^ to selectively deplete phagocytic monocyte/macrophage and DC populations from blood and tissues, resulting in cytokine normalisation, and demonstrating that macrophages contribute to the *in vivo* chronic inflammatory milieu that we observed in R6/2 mice from an early symptomatic stage of disease.

## Results

### Dysregulation of brain and plasma cytokine levels in late stage R6/2 mice

**Figure 1:**
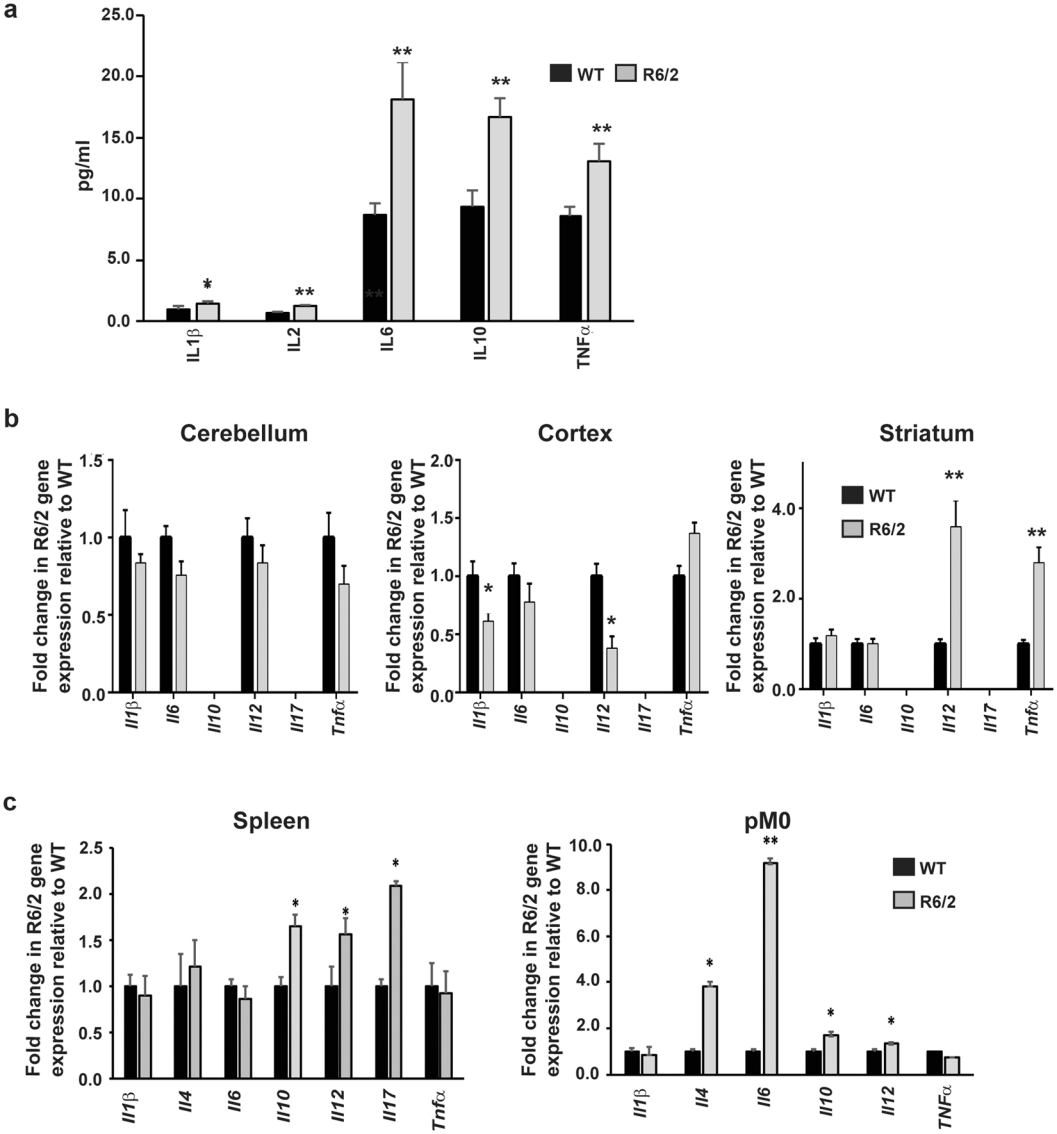
Immune activation in late-stage HD mice as indicated by cytokine levels. (**a**) Cytokine upregulation in the blood of 14 week old R6/2 mice. Plasma levels for R6/2 mice compared to WT are shown ± SEM, (*n* = 20/group IL1β; 16/group IL2; 19/group IL6; 26/group IL10 and 28/group TNFα). (**b**) Increases in cytokine gene expression was limited to the striatum of late-stage R6/2 mice. *Il1β*, *Il2*, *Il4*, *Il6*, *Il10*, *Il12*, *Il17* and *Tnfα* gene expression levels in cerebellum, striatum and cortex of R6/2 mice (*n* = 5/group) compared to WT (*n* = 5/group) ± SEM as measured by qPCR. (**c**) Increased cytokine gene expression in R6/2 splenic and peritoneal macrophage enriched cell populations. R6/2 mouse splenic (*n* = 10/group) and peritoneal macrophage (*n* = 7/group) gene expression for cytokines relative to WT ± SEM quantified by qPCR. *p < 0.05 and **p < 0.01 vs WT by Student’s *t* test. WT = wild type, pM0 = peritoneal macrophages.

Neuroinflammation due to the activation of microglial cells in the brain of HD patients^10^ and mice^25^, as well as the possible activation of hyper-responsive monocytes and macrophages^9,12^, may lead to increased production of cytokines in both the periphery and the CNS of HD subjects. In order to investigate this, we measured plasma and brain cytokine levels by mesoscale discovery (MSD) multiplex ELISA and quantitative real-time PCR (qPCR) respectively. The MSD results revealed a rise in blood IL6 by approximately two-fold in late symptomatic 14 week-old R6/2 mice, while IL1β and TNFα levels were also elevated by just under 50% and IL2 and IL10 increased by over 70% (Fig. 1a).

In the brain, IL6 and TNFα have been documented to have detrimental neurotoxic effects^26–28^. Quantification of cytokine mRNA levels in striatum, cortex and cerebellum showed increased production of *Tnfα* only in the striatum by more than twofold, but there was no increase in *Il6* expression in any of the brain regions tested (Fig. 1b). Furthermore, an approximately threefold increase in *Il12* expression was evident in the striatum of R6/2 mice while the reverse pattern was seen in the cortex, with decreases occurring in *Il12* and *Il1β* by approximately 60% and 40% respectively.

In the periphery, R6/2 splenocyte *Il10*, *Il12* and *Il17* expression levels were at least 50% higher than WT, whereas *Il1β*, *Il4*, *Il6* and *Tnfα* levels remained unchanged. In contrast, cell samples from the peritoneal cavity enriched for macrophages, had approximately four times more *Il4*, and almost ten-fold higher *Il6* than in WT, with more modest increases for *Il10* and *Il12* of approximately 1.8 and 1.3 fold respectively (Fig. 1c). As observed for splenocytes, *Tnfα* and *Il1β* levels were not affected. The results may suggest that blood IL6 upregulation in late stage HD is primarily due to increased macrophage secretion, as increases were only observed in the macrophage enriched samples, and not in splenocytes, where macrophages make up only a small percentage of the cell population. Conversely, the increase in splenocyte *Il12* and *Il10* levels could suggest that cells additional to macrophages, such as helper T cells, are contributing to the increases in the plasma levels of these cytokines.

The cytokine gene expression results for macrophages in the periphery and microglial cells in the brain indicate phenotypically different populations of activated macrophages/microglia in these two compartments, with cells at the periphery exhibiting a more M2-like phenotype, while microglia in the brain favour an M1 phenotype^29^.

### Increased frequencies of activated immune cells in late-stage R6/2 mice

Although the gene expression results for the macrophage enriched peritoneal cells identify myeloid cells as a major source of the upregulated blood cytokines during late stage HD, the possible contribution of other cytokine secreting immune cells such as T lymphocytes and dendritic cells (DC) cannot be completely dismissed. More importantly, some cytokines that were elevated in the periphery in R6/2 mice, such as IL2 in blood and splenic *Il17* mRNA, are not typically secreted by macrophages. This finding prompted us to identify the other cells responsible for the increased peripheral cytokine levels.

**Figure 2:**
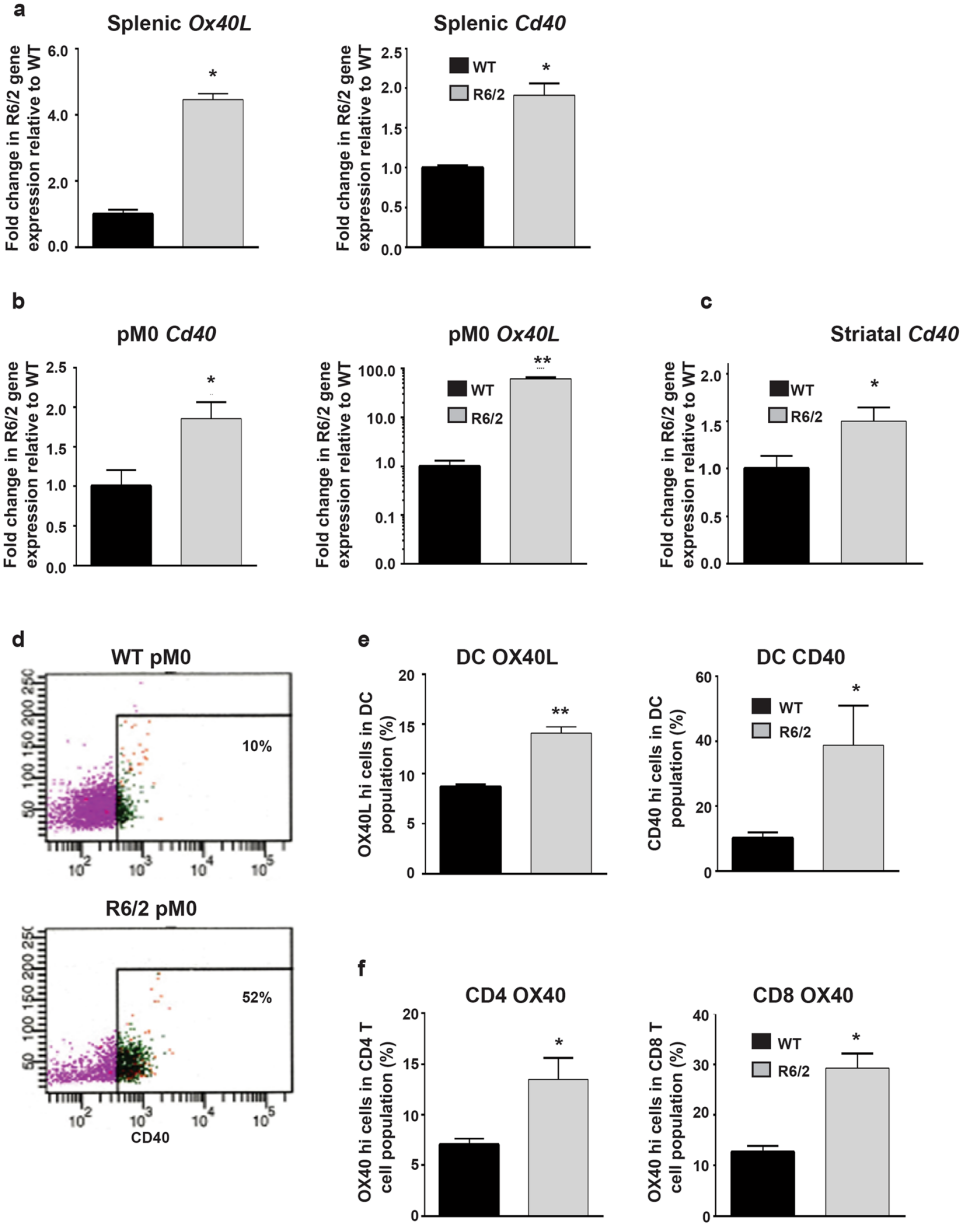
Increased activity of immune cells in late-stage R6/2 mice. (**a**) Increased splenic *Ox40L* and *Cd40* gene expression in late-stage R6/2 compared to WT. Splenic *Ox40L* (*n* = 8/group) and *Cd40* (*n* = 5/group) in R6/2 mice relative to WT ± SEM as measured by qPCR. (**b**) Increased *Cd40* (*n* = 5/group) and *Ox40L* gene (*n* = 5/group) expression in peritoneal macrophages enriched cell populations in late-stage R6/2 compared to WT ± SEM as measured by qPCR. (**c**) Higher *Cd40* expression (n = 5/group) in striatum of 14 week old R6/2 mice vs. WT ± SEM as analysed by qPCR. (**d**) Increased frequencies of activated CD40hi peritoneal macrophages in late-stage R6/2 mice. Percentage of peritoneal macrophages (gated as; CD11b^+^, CD49b^−^, CD8^−^, CD11c^−^, Ly6G^−^) highly expressing cell surface CD40 in 14 week old R6/2 mice and WT as assessed by FACS. The Figure shows a typical example of FACS dot plots obtained for each group analysed. (**e**) Increased frequencies of activated OX40L high and CD40 high splenic DC in late-stage R6/2 mice. Mean percentage ± SEM of DC (gated as; CD11b^+^, CD8^−^, CD11c^+^, Ly6G^−^) highly expressing cell surface OX40L and CD40 in 14 week old WT (*n* = 4/group) and R6/2 mice (*n* = 6/group) as assessed by FACS. (**f**) Increased frequencies of activated OX40 high CD4 and CD8 splenic T cells in late-stage R6/2 mice. Mean percentage ± SEM of T cells (gated as; CD3^+^, CD4^+^ or CD8^+^) highly expressing cell surface OX40 in 14 week old WT (*n* = 5/group) and R6/2 mice (*n* = 6/group) as assessed by FACS. *p < 0.05 and **p < 0.01 vs WT by Student’s *t* test. WT = wild type, pM0 = peritoneal macrophages, DC = dendritic cells.

Following activation, DC and macrophage/microglia upregulate their expression of several activation markers including OX40 ligand (L) and CD40, while, similarly, activated T cells can be identified via the increased expression of cell surface markers such as OX40, CD40L and CD25^30–34^. Gene expression levels for *Ox40L* and *Cd40* in the spleen, peritoneal macrophages and brain regions were measured in order to detect DC and macrophage/microglia activation, and *Ox40* in the spleen to detect T cell activity. In the spleen, which contains T cells and a small percentage of DCs and macrophages, expression of *Ox40L* and *Cd40* were increased in R6/2 mice by approximately four-fold and two-fold respectively (Fig. 2a). Similarly, *Cd40* and *Ox40L* levels were elevated in the peritoneal macrophage enriched cell population (Fig. 2b). Therefore, DC and/or macrophage cell activation can be detected in both spleen and peritoneal macrophages during late-stage HD. Surprisingly, *Ox40* expression did not increase in the spleen (data not shown), suggesting a lack of splenic T cell activity. In the striatum, an increase in *Cd40* expression was also evident (Fig. 2c), which could not be observed in the cortex or cerebellum (data not shown), consistent with the observation of increased cytokine production being confined to the striatum (Fig. 1b). We failed to detect any *Ox40L* expression in any of the brain regions assessed in either WT or R6/2 mice (data not shown).

In order to verify the qPCR results, FACS analysis of OX40L and CD40 expression was performed for (CD11b^+^, CD11c^−^, CD3^−^, Ly6G^−^) splenic and peritoneal macrophages and (CD11b^+^, CD11c^+^, CD3^−^, Ly6G^−^) splenic DCs. Additionally, CD3 and CD4 or CD8 expressing splenic T cells were assessed for OX40 cell surface expression. Figure 2d depicts a representative FACS analysis (n = 5/group) of purified peritoneal macrophages from 14 week old R6/2 mice showing that 52% of the cells are active based on their high expression of CD40 as compared to 10% in WT mice. These results indicate a probable role for macrophages in elevating the plasma cytokine levels during HD. Further assessment of OX40L high expressing activated macrophages showed an increase in their frequency in the peritoneal cavity during late-stage HD (Figure S1).

Assessment of splenic DCs (CD11b^+^ CD11c^+^ CD8^−^ Ly6G^−^) for high expression of OX40L and CD40 by FACS also revealed increases in the numbers of OX40L high and CD40 high cells in late-stage R6/2 mice compared to WT (Fig. 2e). R6/2 mice at 14 weeks of age had almost two fold more activated DCs that were OX40L high, and over three times more activated DCs that were CD40 high than WT.

The FACS analysis of CD3^+^, CD4^+^ helper or CD3^+^, CD8^+^ cytotoxic T cells for high expression of OX40, as a marker for activated T cells, revealed significantly higher percentages of OX40 high expressing CD4 and CD8 T cells in the 14 week old R6/2 mice versus WT. Both cell populations showed approximately twice as many activated cells in their total helper (~7% ± 1 for WT and ~14% ± 3 for R6/2) and cytotoxic (~12% ± 4 for WT and ~30% ± 5 for R6/2) T cell pools in the R6/2 mice (Fig. 2f). These FACS results are contrary to the level of *Ox40* expression, which was not increased in R6/2 spleens as compared to WT.

### Peripheral immune activation precedes immune activity in the brain of R6/2 mice

**Figure 3:**
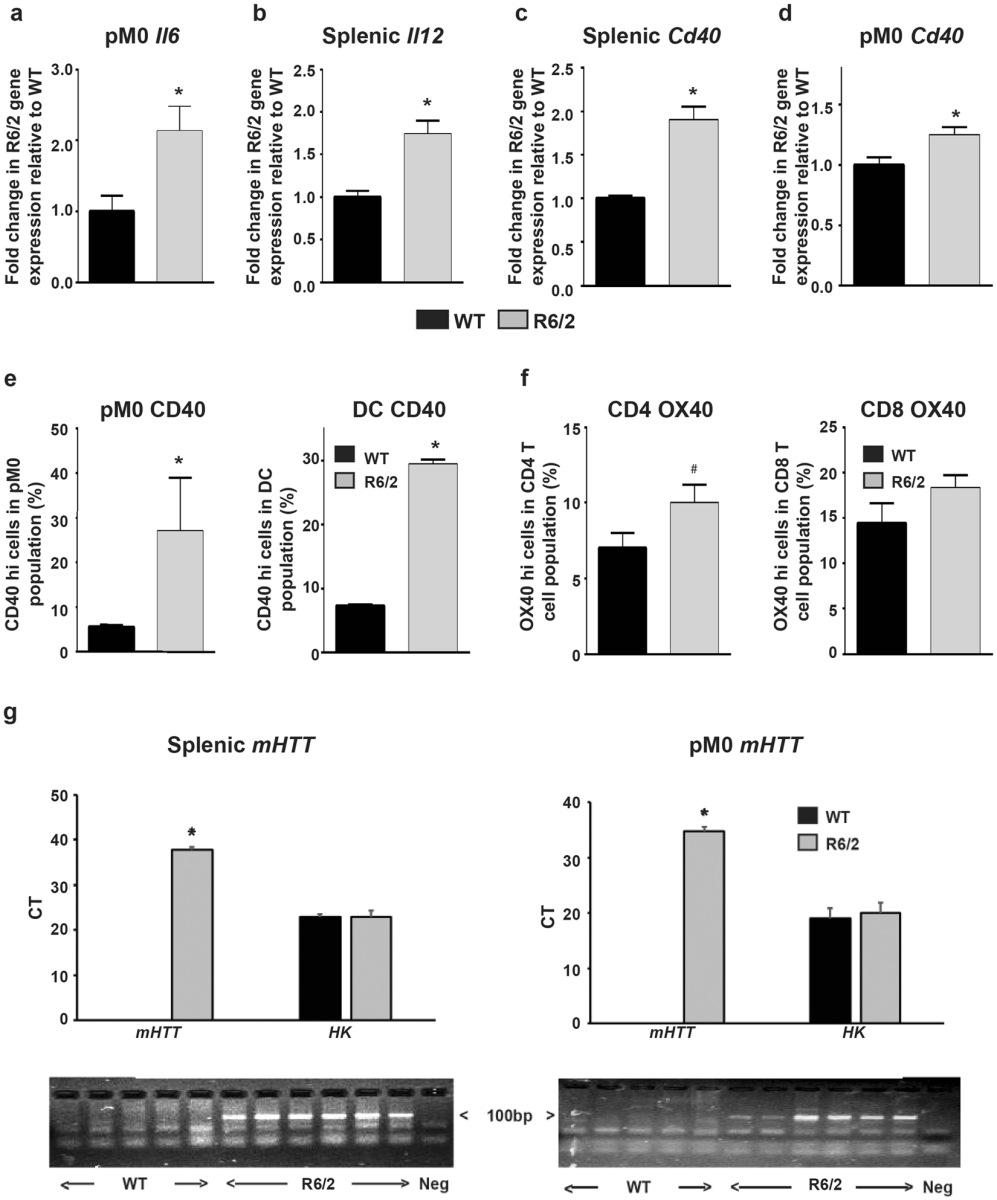
Immune activity in the periphery of early stage R6/2 mice. Upregulated cytokine and activated macrophage cell marker gene expression levels in 8-week-old R6/2 mice. Gene expression of (**a**) *Il6* in peritoneal macrophages (*n* = 10/group), (**b**) *Il12* (*n* = 5/group) and (**c**) *Cd40* in splenic cells (*n* = 5/group), (**d**) *Cd40* in peritoneal macrophages (*n* = 10/group) in R6/2 mice compared to WT. (**e**) Increased frequencies of activated macrophages and DC in early-symptomatic stage R6/2 mice. Mean % ± SEM of peritoneal macrophages (gated as; CD11b^+^, CD8^−^, CD11c^−^, Ly6G^−^) and splenic DC (gated as; CD11b^+^, CD8^−^, CD11c^+^, Ly6G^−^) highly expressing cell surface CD40 in 8 week old WT (*n* = 5/group) and R6/2 mice (*n* = 6/group) as assessed by FACS. (**f**) Increased frequencies of activated T cells in early-symptomatic stage R6/2 mice. Mean percentage ± SEM of splenic T cells (gated as; CD3^+^, CD4^+^ or CD3^+^, CD8^+^) highly expressing cell surface OX40 in 8 week old WT (*n* = 5/group) and R6/2 mice (*n* = 8/group) as assessed by FACS. (**g**) Presence of exon 1 mutant huntingtin (*mHTT*) in splenic mononuclear cells and peritoneal macrophages in 8 week old R6/2 (*n* = 6) but not in WT (*n* = 5) mice as assessed by qPCR, mean CT values ± SEM are shown for both *mHTT* and the geometric mean of *ATP5B* and *B2M* housekeeping genes (HK). Agarose gel electrophoresis of the corresponding *mHTT* qPCR products are shown below each graph. ^#^*p* = 0.06, *p < 0.05 vs WT by Student’s *t* test. WT = wild type, pM0 = peritoneal macrophages.

We have previously observed that the activation of the peripheral immune system, as evidenced by significant increases in a plethora of cytokines in blood, occurs in both HD patients and mouse models from early stages of disease, with macrophages and microglial cells prone to hyperactivity following their *in vitro* stimulation^9,12^. We therefore sought to determine whether immune activity in the R6/2 mouse brain could also be detected at an earlier stage. Therefore, we assessed gene expression and/or protein cytokine levels, and immune activation markers in 8-week-old early symptomatic R6/2 mouse brains, blood and lymphoid tissues/cells. We focused on the cytokines and activation markers that we had found to be upregulated in late stage R6/2 mice. Gene expression analysis showed little change in striatal cytokine expression in R6/2 as compared to WT (data not shown). However, increases in the expression of cytokines and activation markers were identified in R6/2 splenocytes and/or peritoneal macrophages by 8 weeks of age. *Il6* was increased in the peritoneal macrophage enriched cell sample by over two fold (Fig. 3a), splenic *Il12* was increased by over 70% (Fig. 3b), *Cd40* was increased in the spleen by over 90% (Fig. 3c) and in peritoneal macrophages by approximately 25% (Fig. 3d).

Quantification of protein levels in blood from 8-week-old R6/2 mice showed no increase in IL6, IL10, IL1β and TNFα cytokines levels (data not shown), despite the apparent increased gene expression for some of these cytokines within peripheral cell populations. This finding suggests that the immune system has not been activated for long enough to allow cytokines to accumulate. In contrast, the cell surface expression of CD40 on macrophages and DCs mirrors the gene expression findings: with a 20% increase in the percentage of activated macrophages and approximately 30% increase in the percentage of activated DCs, indicating that these cells may have become recently activated (Fig. 3e).

The percentages of OX40 high expressing cells were increased for the CD4 T cells (*p* = 0.06) but not for the CD8 T cell population, indicating that helper T cells are on the cusp of activation at this stage of disease (Fig. 3f). Interestingly, unlike for *Cd40* (Fig. 3c,d), *Ox40L* gene expression, and cell surface protein expression on macrophages and DCs remained similar to WT levels (data not shown). This further supports the notion that we have analyzed these cell populations at the early stages of immune activity, since OX40L upregulation on antigen presenting cells (APC) usually occurs at a later stage of activation, after CD40 surface expression increases^33,34^. Furthermore, FACS assessment of peritoneal macrophages from 8-week-old mice also revealed similar results with increases in the percentages of CD40 highly expressing cells in the R6/2 mice versus WT controls (Fig. 3e) but with no differences in OX40L highly expressing peritoneal macrophage frequencies in the R6/2 and WT groups (Figure S2).

Assessment of exon 1 mutant *HTT* gene expression within R6/2 peritoneal macrophages and splenic mononuclear cells, containing lymphocytes, macrophages and DC, confirmed that the R6/2 transgene was expressed in these cells and as expected was absent from their WT counterparts (Fig. 3g).

### Late stage *z*Q175 mice recapitulate the immune activation characteristics observed in 14-week-old R6/2 mice

**Figure 4:**
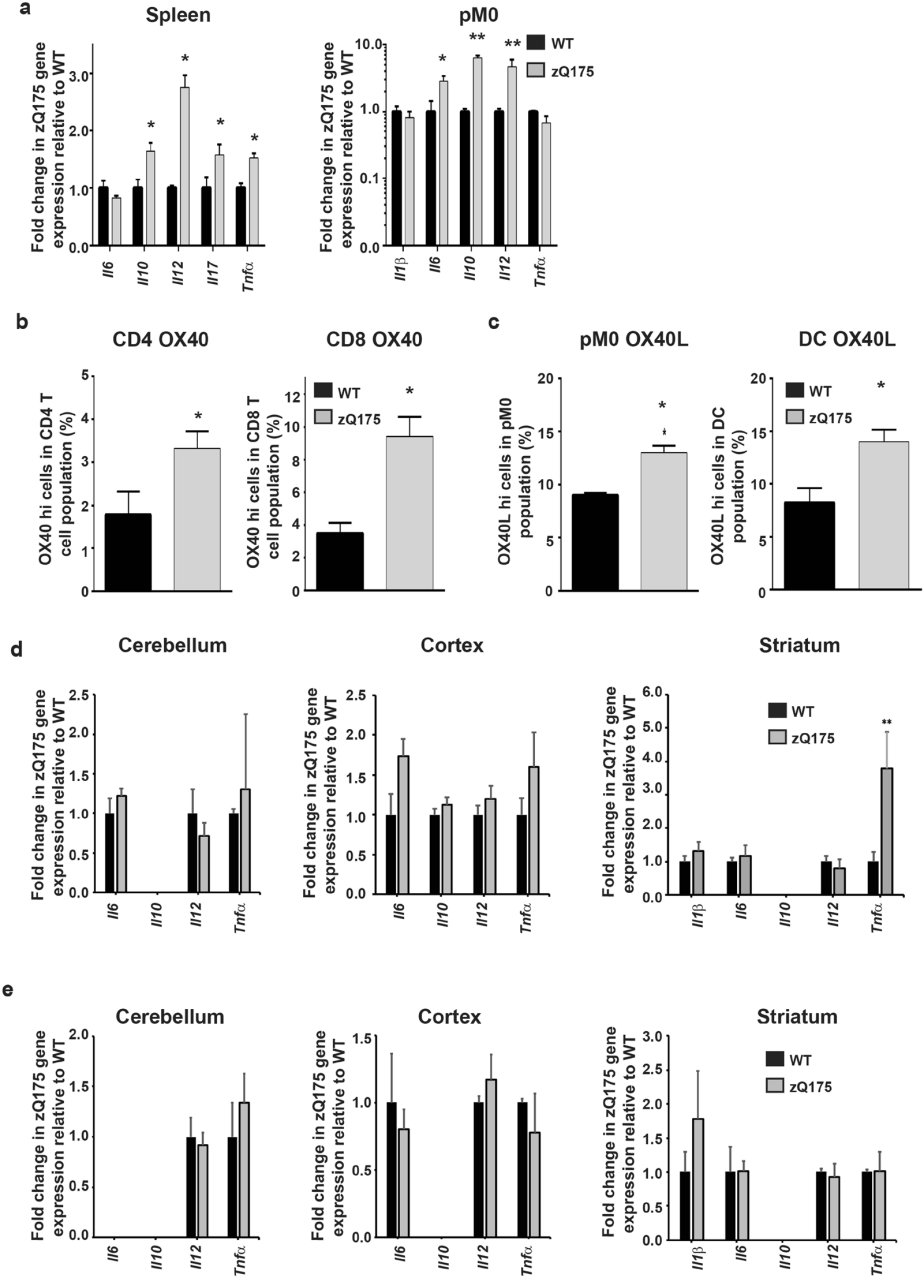
Immune activation in zQ175 mice. (**a**) Increased gene expression in late-stage zQ175 mice for several cytokines in splenic (*n* = 5–6/group) and peritoneal (*n* = 5–6/group) macrophage populations. 22-month-old zQ175 mice ± SEM splenic and peritoneal macrophage gene expression for cytokines relative to WT as quantified by qPCR. (**b**) Increased activated T cell numbers in 22-month-old Q175 mice. Mean % ± SEM of splenic T cells (gated as; CD3^+^, CD4^+^ or CD8^+^) expressing OX40 highly in 22 month old WT (*n* = 5/group) and zQ175 mice (*n* = 6/group) as assessed by FACS. (**c**) Increased activated peritoneal macrophage and DC frequencies in 22-month-old zQ175 mice. Mean percentage ± SEM of peritoneal macrophage and splenic DC expressing OX40L highly in 22 month old WT (*n* = 5/group) and zQ175 mice (*n* = 5/group) as assessed by FACS. (**d**) Increases in cytokine gene expression was limited to the striatum of 12 month old zQ175 mice. Mean *Il1β*, *Il6*, *Il10*, *Il12* and *Tnfα* gene expression levels in cerebellum, striatum and cortex of zQ175 mice (*n* = 5/group) compared to WT (*n* = 5/group) ± SEM as measured by qPCR. (**e**) There were no increases in cytokine gene expression in 21 month old zQ175 mice. Mean *Il1β*, *Il6*, *Il10*, *Il12* and *Tnfα* gene expression levels in cerebellum, striatum and cortex of zQ175 mice (*n* = 5/group) compared to WT (*n* = 5/group) ± SEM as measured by qPCR. *p < 0.05 and **p < 0.01 vs WT by Student’s *t* test. WT = wild type, pM0 = peritoneal macrophages, DC = dendritic cells.

In order to confirm the relevance of phenotypes identified in R6/2 mice, we ascertained whether these also develop in a knock-in HD mouse model, which more closely recapitulates the genetic basis of HD. The expression levels of cytokines in splenocytes from late-stage zQ175 mice at 22 months of age showed similar increases to those observed in late-stage R6/2 mice: *Il10* by over 50%, *Il12* by almost three-fold, and *Il17* by approximately 50%. In contrast to R6/2 mice, *Tnfα* was also increased (Fig. 4a). In the peritoneal macrophage enriched cell populations, *Il6* gene expression was almost three-fold higher, *Il10* over six-fold higher and *Il12* around five-fold higher in the zQ175 mice versus WT (Fig. 4a). These results indicate that the M1 activated macrophage phenotype is more evident in zQ175 than in R6/2 mice, as indicated by a greater increase in *Il12*, as well as the upregulation of *Tnfα* expression in spleen. This suggests that the zQ175 mice have a less stringent M1 or M2 macrophage response. The frequencies of activated T cells, macrophages and DCs in the spleen or peritoneal cavity were also upregulated in late-stage *z*Q175 mice as compared to WT, according to the cell surface expression of activation markers: OX40 (Fig. 4b) and OX40L (Fig. 4c) respectively, which were increased. Analysis of cytokine gene expression in brain regions of zQ175 mice at 12 months of age revealed that only *Tnfα* expression was increased in the striatum, as had been the case for R6/2 mice. However, unlike R6/2 mice, *Il12* was not upregulated in the striatum, and nor was it decreased in the cortex of zQ175 mice (Fig. 4d). We were unable to observe any increase in striatal cytokine levels in late stage 21 month old zQ175 mice (Fig. 4e), which may be due to the effect of immune exhaustion in these old zQ175 mice as a result of chronic immune activation of self-renewing HD microglial cells^35^.

### *In vivo* depletion of macrophages and DC via clodronate liposome infusion normalizes cytokine levels during late stage HD

**Figure 5:**
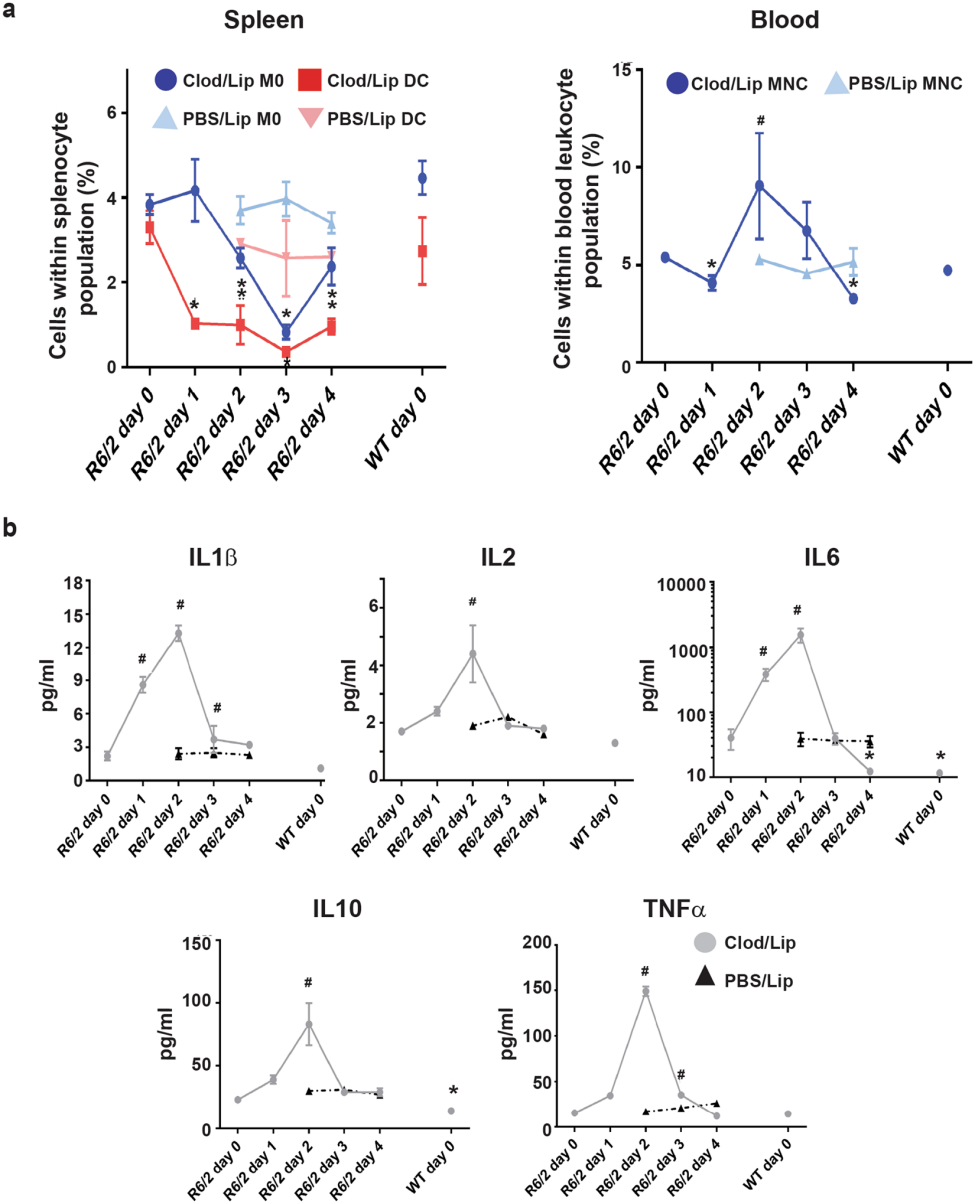
Clodronate liposome depletion of splenic macrophage and DC results in reduced IL6 levels in R6/2 mice. (**a**) IV clodronate liposome treatment of late-stage R6/2 mice depletes splenic macrophages (gated as; CD11b^+^, CD8^−^, CD11c^−^, Ly6G^−^) and DC (gated as; CD11b^+^, CD8^−^, CD11c^+^, Ly6G^−^), and monocytes (gated as; CD11b^+^, CD8^−^, CD11c^−^, Ly6G^−^). Mean percentage ± SEM of splenic macrophages and DC, and monocytes in the total leukocyte population following clodronate liposome or PBS liposome injection into late-stage R6/2 mice as analyzed by FACS. (**b**) Clodronate liposome treatment only normalises IL6 to WT plasma levels in late-stage R6/2 mice. Mean concentration of TNFα, IL1β, IL2, IL6 and IL10 ± SEM following clodronate liposome or PBS liposome injection as assessed by MSD. *p < 0.05 decrease, ^#^*p* < 0.05 increase as compared to R6/2 day 0, (*n* = 5/group). WT = wild type, IV = intravenous, DC = dendritic cells, M0 = macrophages. MNC = monocytes, Clod/lip = clodronate liposome, PBS/lip = PBS liposome.

Our current and previous results^13^ strongly implicate macrophages as the major contributors to the immune dysregulation that occurs in HD. In order to ascertain the role of these cells, we utilized the toxic clodronate immunosuppressive drug, commonly used for the treatment of osteoporosis^36^. In order to specifically deplete monocyte and macrophage cells *in vivo*, clodronate packaged in liposomes were administered via IV or IP injection into R6/2 mice at 13–14 weeks of age^37–39^. IV injection resulted in the depletion of (CD11b^+^ CD8^−^ CD11c^−^ Ly6G^−^) blood monocytes and splenic macrophages, and (CD11b^+^ CD8^−^ CD11c^+^ Ly6G^−^) splenic DCs, in both the blood and spleen of R6/2 mice (Fig. 5a) and WT (data not shown), but not T cells or neutrophils (data not shown). PBS liposome treatment had no impact on the frequencies of blood monocytes, and splenic macrophages and DCs (Fig. 5a). Depletion of splenic DCs and splenic macrophages was achieved one and two days post clodronate injection respectively, with maximal depletion of around 75% for macrophages and almost 90% of DCs occurring on the third day, this being maintained until day four post injection. Blood monocyte depletion was observed earlier, one day after clodronate injection. The recovery of blood monocytes two days post injection may indicate a response by the bone marrow to replete monocyte numbers, while its subsequent gradual reduction from day two may be due to the recruitment of monocytes into tissues, in order to replenish the diminished macrophage numbers post clodronate liposome depletion^24,39^.

Analysis of plasma cytokine levels following clodronate liposome treatment revealed that the drug initially promoted a gradual increase in all of the cytokines assessed (Fig. 5b). This peaked two days post injection, rising from approximately 20 pg/mL to over 80 pg/mL for IL10, from 2 pg/mL to 13 pg/mL for IL1β, by over 60% for IL2, by approximately 130 pg/mL for TNFα and by over fifty-fold for IL6 (Fig. 5b). No significant changes were evident in the PBS liposome treated control mice at day 2 and day 4 post injection (Fig. 5b). This observation can be explained by the activation of the immune system by dead or dying monocytes and macrophages, increasing cytokine production by immune cells, which includes any remaining monocytes and macrophages^40^. Such initial, dramatic cytokine spikes could potentially have detrimental effects, however no adverse effects were observed up to ten days post drug injection. At day 3 post injection, when maximum splenic macrophage and DC depletion was observed, most of the cytokines returned to basal levels, while at day 4 post injection IL6 levels had significantly decreased from that observed prior to injection. It is worth noting, that of the cytokines assessed here, only *Il6*, and *Il10* levels were elevated in the peritoneal macrophage enriched cell population of late stage R6/2 mice compared to WT (Fig. 1c). Therefore, the greater likelihood of a reduction in IL6 and IL10, but not TNFα, IL1β or IL2 post macrophage depletion, was perhaps to be expected. However, the lack of a reduction in IL10 levels suggests that other cells, such as T cells and B cells, may upregulate this cytokine in order to compensate for the loss of macrophage IL10 production following the depletion of these cells^41,42^. Our inability to detect any differences in plasma basal levels in R6/2 compared to WT mice for the other upregulated cytokines (Fig. 1a) is likely due to the low sample size for this experiment. The detection of differences for only plasma IL6 and IL10 (Fig. 5b), the cytokines that show the greatest disparity between R6/2 and WT at 14 weeks of age would support this conclusion.

In order to resolve the limitation of a small sample size, we repeated the clodronate liposome infusion study twice and pooled the experimental results. However, the splenic macrophage and DC depletion dynamics were somewhat variable between experiments. Maximal splenic macrophage (decreasing to 0.8–0.9% CD11b^+^ CD8^−^ CD11c^−^ Ly6G^−^ cells from 3.9–4.5% prior injection) and DC depletion (decreasing to 0.1–0.2% CD11b^+^ CD8^−^ CD11c^+^ Ly6G^−^ cells from 0.6–0.8% prior injection) was achieved in all experiments, but occurred at different time-points, between one to three days post injection (data not shown). This difference may be due to batch-to-batch drug variations. Despite the difference in depletion timing, macrophage frequencies and cytokine levels did not differ significantly at the start of the experiment, or at the time when highest cell depletion was seen between individual experiments.

**Figure 6:**
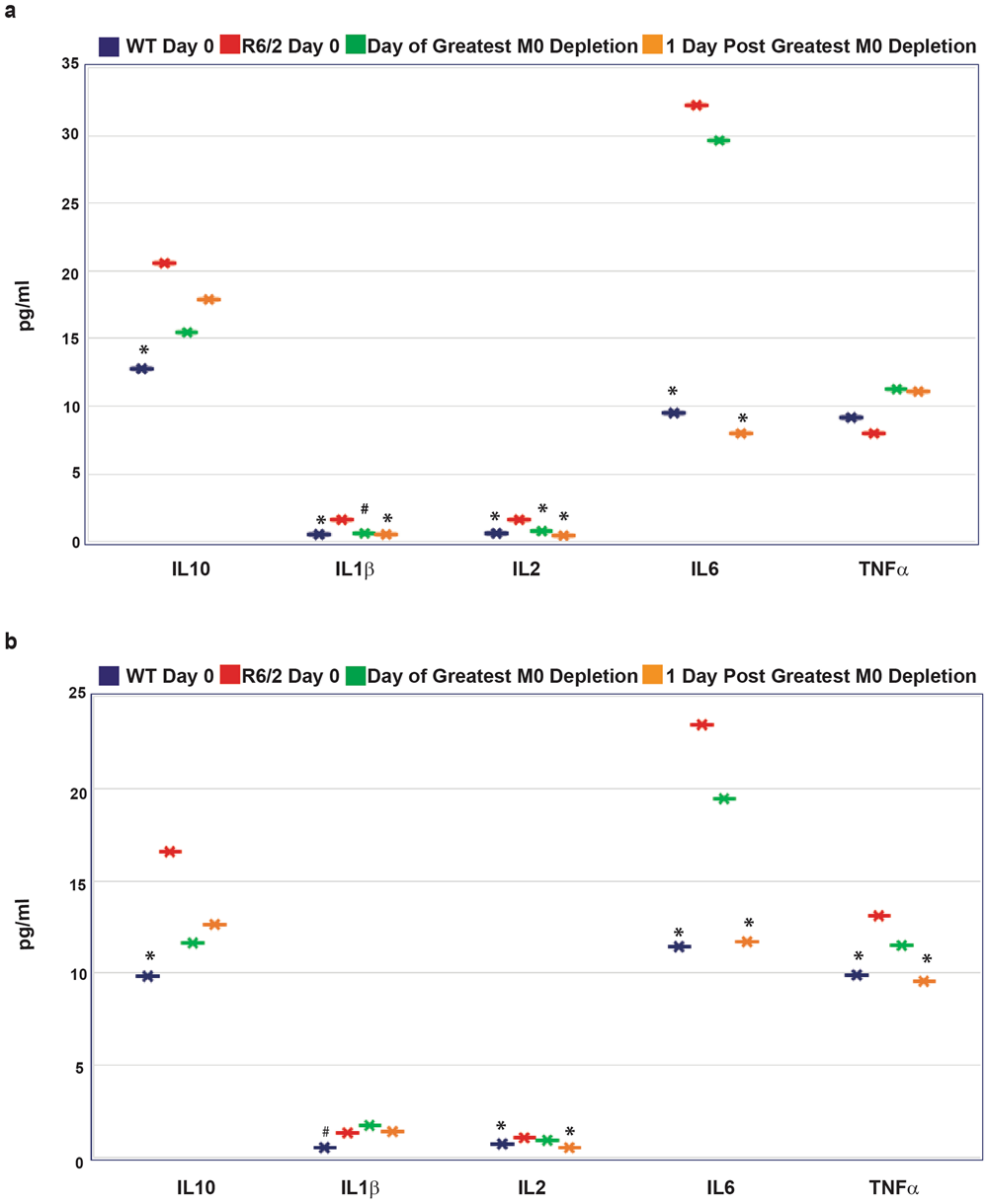
Clodronate liposome normalisation of cytokine levels in late-stage R6/2 mice. (**a**) Clodronate liposome treatment reduced IL1β, IL2 and IL6 levels in late-stage R6/2 mice post IV treatment (*n* = 8–12/group). Mean concentration of IL10, IL1β, IL2, IL6 and TNFα ± SEM following clodronate liposome IV injection, at the day of greatest splenic M0 depletion and one day later as assessed by MSD. (**b**) Clodronate liposome treatment normalised TNFα, IL2 and IL6 levels in late-stage R6/2 mice post IP treatment (*n* = 9–12/group). Mean concentration of TNFα, IL1β, IL2, IL6 and IL10 ± SEM following clodronate liposome IP injection, at day of highest splenic M0 depletion and one day later as assessed by MSD. All comparisons to R6/2 levels at day 0, ANOVA with Bonferroni correction. *p < 0.05, ^#^*p* = 0.06. WT = wild type, IV = intravenous, IP = intraperitoneal, M0 = splenic macrophages.

Therefore, we decided to look specifically at the cytokine levels prior to treatment, for all three experiments, on the day when maximal macrophage depletion was achieved and on the day following maximal macrophage depletion. Our combined results not only confirmed the significant reductions in IL6 levels at the day following maximal macrophage depletion, but additionally, revealed decreases in IL1β and IL2, not only at this time-point, but also on the day of maximal splenic macrophage depletion (Fig. 6a). The reduction of IL1β and IL2 one day ahead of IL6, may be explained by their shorter half-life when compared with IL6^43–45^, thus resulting in their more rapid loss post significant macrophage depletion.

Next, we assessed whether clodronate injection via the IP route produced similar results to those of IV injected mice. An IP injection will result in macrophages and DCs within tissues being depleted without affecting blood monocytes, and will reveal whether this is sufficient to account for the effect of clodronate liposomes on R6/2 cytokine levels^46^. The dynamics of cell depletion in the spleen post IP injection were variable, similar to those seen by IV. The maximal depletion of macrophages (from 4% ± 0.3% to 1.1% ± 0.4%) and DCs (0.4% ± 0.09% to 0.2 ± 0.04%) were at comparable levels to those for IV, but occurred later, on days three and four post injection, as expected, there was no effect on blood monocyte numbers (data not shown). Plasma cytokine levels were measured by MSD multiplex analysis on the day when maximal cell depletion was achieved, as well as the following day. IL6 levels were decreased one day after maximum splenic macrophage depletion, but there was no effect on IL1β as had been observed for the IV injected mice. This may be due to IL1β being predominantly secreted by blood monocytes, which remain intact post IP clodronate treatment or, to the greater macrophage depletion within spleens via the IV route compared to the IP route (data not shown). However, TNFα levels were also reduced following cell depletion, which had not occurred after IV injection (Fig. 6b). Taking the IP and IV results together, these findings suggest that reductions in specific cytokines post clodronate treatment only occur if these cytokines are at higher levels in R6/2 plasma as compared to WT at treatment initiation. The results indicate that depletion of macrophages and DCs within the spleen, and probably other tissues, is sufficient to prevent the increased plasma cytokine levels in R6/2 mice, suggesting that macrophages and/or DCs are central to the observed increases in IL1β, IL2, IL6, and TNFα levels.

## Discussion

Herein we have shown that the cells that promote immune hyperactivation during disease progression in HD mouse models are not only macrophages and monocytes, as inferred by previous studies, but also T cells and DCs. We additionally characterised the macrophage response in HD according to the M1 and M2 phenotypic classification, and found that this differed depending on the tissue in which these cells reside. In R6/2 mice, within the brain, microglial cells took up a predominantly M1 cytotoxic profile, whilst in the periphery, macrophages favour an M2 humoral/repair phenotype^29^. Thus, while microglial cells produced elevated *Il12* and *Tnfα* but lacked *Il10* and *Il6* increases, their peripheral counterparts increased their production of *Il6* and *Il10* as well as the M2 promoting cytokine *Il4*, but lacked *Tnfα* upregulation. This indicated a phenotypic discordance in the activated macrophage cells populating these two distinct regions. Although the M1/M2 paradigm has been traditionally used to identify seemingly two phenotypically distinct macrophage populations generated following their activation, such differences have been shown to be less clear cut more recently^47,48^. In keeping with this, in the zQ175 mice the M1/M2 paradigm was less evident, but nevertheless some remnants of a peripheral M2 peritoneal macrophage and an M1 like microglial response could still be seen.

The absence of *Il10* upregulation within the brain despite, its increases in the periphery, not only support the idea that microglial cells are predominantly M1 in phenotype but may also indicate that these cells are not trying to counter the inflammation within the CNS, at least via the secretion of anti-inflammatory IL10. Interestingly, the study by Bjorkvist *et al*. in three different HD mouse models, not only saw similar increases in the levels of blood M2 type cytokines, IL6 and IL10 in the periphery but also found no significant increases in the M1 promoting cytokine IFNγ and, unlike in our study, TNFα as well, thus further supporting the notion that activated peripheral monocytes/macrophages show an M2 dominant phenotype^9^.

Inflammation and immune activation has been reported to be a feature of HD and our analysis of cytokine levels in the brain and plasma of late-stage R6/2 and zQ175 mice further confirmed this finding. Of particular interest, this included increases in IL6 and TNFα, which are known to have neurotoxic properties^26,28^ and have the capacity to cross the blood brain barrier^49,50^. Interestingly, TNFα expression was increased in the brain and elevated levels of this cytokine were found in the blood, despite no increase in *Tnfα* gene expression in the peripheral immune compartments assessed. Therefore, TNFα may have migrated from the brain to the blood. However, other peripheral TNFα sources such as lymph nodes cannot be discounted as the possible source for the raised blood TNFα levels.

IL17 has been found to be secreted by astrocytes within the brain^51^, however we failed to detect *Il17* gene expression in either WT or R6/2 mice in our qPCR analysis which might suggest a minimal role of these glial cell population to the observed cytokine upregulation in the HD brain, since they are also coincidently capable of secreting cytokines such as TNFα^52^. Clearly, determining the mechanisms for the observed site-specific differences in HD brain cytokine production requires further elucidation.

Subsequent analyses to quantify the frequencies of activated immune cells, responsible for the upregulated cytokines, revealed not only increases in the levels of active macrophages, as predicted, but also DCs and T cells. These results indicate a possible contribution of these cells to the macrophage-induced inflammation seen during HD. Previously, we failed to detect any increases in activated macrophage frequencies based on their MHCII cell surface expression^13^. This result is particularly interesting given that unlike M1 macrophages, M2 macrophages do not notably upregulate MHCII on their surface post-activation. This not only provides an explanation for the previous results but also strengthens our current findings, suggesting that peripheral macrophages predominantly adopt an M2 phenotype in R6/2 mice^29,53^. Late-stage zQ175 mice revealed similar increases in cytokine production, and activated immune cells, to their 14-week-old R6/2 counterparts. However, these mice were found to have a more balanced M1 and M2 macrophage response, which may be due to age differences or minor differences in disease presentation between the two models. We previously reported that the NF*κ*B transcription factor is dysregulated in HD patient monocytes/macrophages^13^. It would be interesting to assess whether any of the other HD murine cell populations we have found to have enhanced activation levels also utilise NFκB for their activity.

The analysis of immune activity during specific stages of disease progression in R6/2 mice provided an insight into the initiation of immune activation. Initial signs of immune activity in macrophage and DC populations occurred at an early symptomatic stage, at which there was no immune activity in the striatum, a brain region that shows immune dysregulation during late-stage disease. Our finding that immune activation in the periphery precedes that in the brain could suggest that the peripheral immune system may, at least in part, initiate or enhance the activation of brain microglia, as previously proposed by our group and others^9,11^, possibly through the secretion and provision of IL6 and other blood brain barrier penetrating cytokines. These cytokines may stimulate microglia directly, or indirectly, to become activated e.g. through neuronal cell killing, as could be the case for IL6^54^.

Clodronate liposome depletion of macrophages and DCs *in vivo*, resulted in the normalisation of cytokine levels in late-stage R6/2 mice, and provided the first *in vivo* evidence to confirm that these APCs upregulate their cytokine levels. Our results provide a valuable insight into the impact of the dysregulated macrophage and DC responses on the HD immune system, identifying these cells as major accomplices in promoting the chronic *in vivo* inflammation that manifests as excessive blood cytokine content in HD mice. Follow up studies would be required to decipher the effect of peripheral cytokine normalisation on HD associated neuroinflammation and ultimately, HD disease pathology. From our experience as well as from previous immunomodulatory HD studies, this would require consistent, long term and continuous blood cytokine reduction. Thus multiple, scheduled clodronate treatments would be required to prevent the observed recovery of monocytes/macrophages and DC numbers in order to investigate the impact of prolonged, sustained drug induced macrophage and DC depletion on disease- related phenotypes. Several immunomodulatory regimens, which have achieved long-term immunosuppression in HD mice, have however conferred beneficial effects in improving symptoms and/or survival. We propose that such effects are, at least in part, due to their impact on macrophages and/or DCs, be it upstream, as in the case of bone marrow transplantation, where HD myeloid cells are replaced by wild type cells^14^, or downstream, where specific macrophage-upregulated cytokines are inhibited, such as peripheral IL6 neutralization and TNFα signalling inhibition^15,16^. Although we show that clodronate liposome therapy significantly dampens mutant huntingtin induced macrophage hyperactivity, and elevated cytokine production, the long-term use of such a potent drug may be deleterious, considering the important role of macrophages in other biological functions such as red blood cell recycling^55^. Thus, more defined therapeutic strategies targeting the production of specific cytokines, or biasing their M1/M2 activation phenotype, are likely to have more desirable outcomes. Nevertheless, herein, we have shown that macrophages and DCs play a protagonist role in the immune hyper-activation and chronic inflammation associated with HD *in vivo*, providing a case for immunomodulatory regimens targeting these particular cells as potential therapeutic strategies for this disease.

## Methods

### Mice

Hemizygous R6/2 mice were generated by backcrossing R6/2 males to (CBA x C57BL/6) F1 females (B6CBAF1/OlaHsd, Harlan Olac, Bicester, UK)^21^. Heterozygous zQ175 knock-in mice^18^ were supplied from CHDI colonies (Jackson Laboratory, Bar Harbor, ME, USA) and maintained by backcrossing to C57BL/6J (Charles River). All animals had unlimited access to food and water, and were kept in sterile housing conditions in individually ventilated cages with environmental enrichment as previously described^56^. Mice were subject to a 12-h light/dark cycle. All experimental procedures were performed in accordance with Home Office regulations and approved by the King’s College London and University College London Ethical Review Committees.

R6/2 and zQ175 mice were genotyped and the CAG repeat was measured as previously described^57^. The mean repeat size (±SD) for all mice used in the study was 180 ± 10 for zQ175 mice and 205 ± 7 for R6/2 mice. Mice were euthanized via CO_2_ asphyxiation or cervical dislocation as required. Equal numbers of male and female R6/2 and zQ175 mice, and their WT littermate controls were used unless stated otherwise.

### Blood, plasma, tissue and cell sample collection

Blood was obtained via tail vein puncture followed by decapitation post cervical dislocation and collected into EDTA tubes. Plasma was obtained by spinning blood at 2000 × *g* for 5 min obtaining the top plasma layer, while the bottom layer, containing blood cells, was used for subsequent FACS analysis. Following euthanasia, whole brains, striatum, cortex, cerebellum and whole spleens tissues were dissected. Brain samples were snap frozen in liquid nitrogen immediately after dissection and stored at −80 °C until required. Single cell suspensions from spleen samples were obtained as described previously^58^, in some cases mononucleated cells from isolated splenocytes were purified by density gradient centrifugation using Histopaque (Sigma). Peritoneal macrophages (pM0) were isolated as described previously^12^. All cell samples were treated with red blood cell lysis solution (R&D systems) in order to remove red blood cells prior to subsequent processing.

### Plasma analyses

Cytokine (IL1β, IL2, IL6, IL10, IL12 and TNFα) levels in plasma were quantified using Meso Scale Discovery (MSD) assays as per the manufacturer’s protocol and analysed on a SECTOR 2400 instrument (MSD).

### RNA extraction and quantitative real time gene expression analysis

Total RNA from cell sample was extracted with the mini-RNA kit according to the manufacturer’s instruction (Qiagen). Reverse transcription (RT) was performed using the MMLV Superscript reverse transcriptase (Invitrogen) and random hexamers (Operon) as described previously^59^. All Taqman quantitative real-time PCR (qPCR) reactions were performed using the Chromo4 real-time PCR detector (Biorad) and quantified via the 2^−ΔΔCT^ method^60^. Gene expression for the gene of interest was normalised to the geometric mean of endogenous housekeeping genes (beta 2 microglobulin and *Atp5b* for peripheral tissue/cells or *Ubc* and *Atp5b* for brain tissue) for each sample. Primer and probe sets for genes of interest (*Il1β*, *Il2*, *Il4*, *Il6*, *Il10*, *Il12*, *Il17*, *Tnfα*, *Cd40*, *Ox40 and Ox40L*) and housekeeping genes were purchased from Biorad. For mutant huntingtin gene expression analysis: forward primer sequence = 5′gctgcacctaccgtgagt3′, reverse primer sequence = 5′cgcaggctgcagggttac3′, and FAM labelled probe sequence = 5′agctccctgtccgggcgg3′ were used.

### Flow cytometry (FACS)

For detecting macrophage/monocytes, DCs, neutrophils^61^ and CD8 T cells^62^, 0.5 × 10^6^ cells were stained with anti-mouse CD8-FITC, anti-mouse CD49b-FITC, anti-mouse CD11b-phycoerythrim (PE), anti-mouse CD11c-APC-Cy7 (for DC exclusion) or CD11c-Per-CP (for activated DC population analysis) and anti-mouse Ly6G-APC-Cy7. To detect activated macrophage and DC populations, anti-mouse CD40-APC or OX40L-APC antibodies were also added (all from eBioscence). To detect activated T cells, anti-mouse CD3-PE, anti-mouse CD4-PerCP, anti-mouse CD8-FITC and anti-mouse OX40-APC or anti-mouse CD25-APC antibody (all from eBioscience) staining was performed for 30 min at 4 °C after blocking with 10% FCS in PBS with 10 mM EDTA, then fixed with 2% paraformaldehyde in PBS with 10 mM EDTA. Cells were analysed using FACS Canto (Becton Dickinson) acquiring 20,000 leukocytes (gated according to forward and side scatters with doublets excluded according to FCS-A/FCS-H dot plots). FACS results were subsequently analysed with FACS Diva (Becton Dickinson). Corresponding unstained cell samples for each antibody were acquired to establish gating areas for positively staining cells.

### *In vivo* clodronate liposome treatment

13 to 14 week old female mice were injected intravenously (IV) via the tail vein or intraperitoneally (IP) with 200 µl or 400 µl clodronate liposome (Liposoma) at 5 mg/mL respectively or with control liposomes containing PBS. Preliminary dosing studies were performed to determine the optimal dose required to deplete target cells over ten days. Body temperature was monitored post injection, and no adverse effects were seen. Mice were sacrificed by cervical dislocation at days 1, 2, 3 and 4 post treatment for IV injected mice or 3, 4, 5 and 6 post treatment for IP injected mice as well as day 0 (prior injection). Blood, spleen and liver samples were obtained as described above for plasma cytokine and/or cell (monocyte/macrophage, DC, CD8 T cells or neutrophil) content quantification by MSD or FACS respectively as detailed above.

### Statistical Analysis

Differences between specified groups were detected using one-way ANOVA with Bonferroni correction (SPSS software) or Student’s *t*-test (Microsoft Excel software) as stated in the Figure legends. *P-values* of 0.05 or less were considered significant.

## Electronic supplementary material

Supplementary information
