# Supplementary material for: In vivo neutralization of the protagonist role of macrophages during the chronic inflammatory stage of Huntington’s disease
Source: Sci Rep. 2018 Jul 30;8:11447. doi: 10.1038/s41598-018-29792-x (PMC6065433; doi:10.1038/s41598-018-29792-x)
Supplement: Supplementary file 1 — Supplementary information [file 41598_2018_29792_MOESM1_ESM.pdf]

In vivo neutralization of the protagonist role of macrophages during the chronic inflammatory stage of Huntington's disease

Jeffrey Pido-Lopez, Ralph Andre, Agnesska C. Benjamin, Nadira Ali, Sahar Farag,

Sarah J. Tabrizi and Gillian P. Bates.

Huntington's Disease Centre, Department of Neurodegenerative Disease and Dementia Research Institute, UCL Institute of Neurology, University College London, London WC1N 3BG, UK.

Corresponding author: j.pido@ucl.ac.uk or gillian.bates@ucl.ac.uk

Supplementary Figures

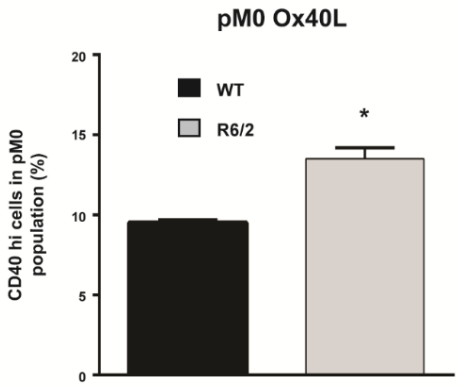

Figure S1. Increased activated pM0 frequencies in late-stage R6/2

Increased activated pM0 frequencies expressing Ox40L in late-stage R6/2 (*n* = 6) mice compared to age-matched WT (*n* = 6) mice as assessed by FACS. **p* <0.05 by Student's test.

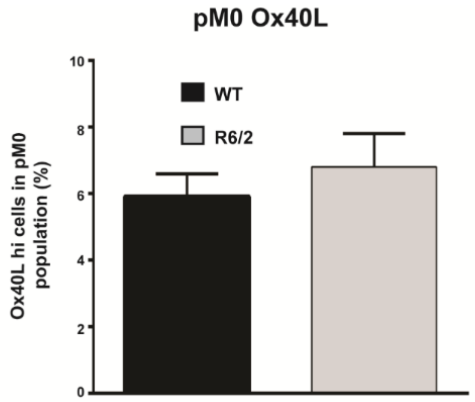

Figure S2. Increased activated pM0 numbers expressing Ox40L in early symptomatic R6/2

Increased activated pM0 numbers expressing Ox40L in 8 week old R6/2 (*n* = 5) mice compared to age-matched WT (*n* = 6) mice as assessed by FACS.
